# Early treatment response to piperacillin/tazobactam in patients with bloodstream infections caused by non-ESBL ampicillin/sulbactam-resistant *Escherichia coli*: a binational cohort study

**DOI:** 10.1007/s15010-023-02074-z

**Published:** 2023-07-18

**Authors:** Selma Tobudic, Christina Bahrs, Lisa Schneider, Emilia Paulussen, Lucie Bartonickova, Stefan Hagel, Peter Starzengruber, Heinz Burgmann, Mathias W. Pletz

**Affiliations:** 1 Division of Infectious Diseases, Department of Internal Medicine I, Medical University Vienna, Vienna, Austria; 2 Institute of Infectious Diseases and Infection Control, Jena University Hospital/Friedrich-Schiller-University, Am Klinikum 1, 07747 Jena, Germany; 3 Institute of Medical Microbiology, Jena University Hospital/Friedrich-Schiller-University, Jena, Germany; 4 Division of Clinical Microbiology, Department of Laboratory Medicine, Medical University of Vienna, Vienna, Austria; 5 Center for Sepsis Care and Control, Jena University Hospital/Friedrich-Schiller-University, Jena, Germany

**Keywords:** Gram-negative bacteraemia, Ampicillin/sulbactam resistance, Piperacillin/tazobactam, Early treatment response, 28-day mortality, Relapse

## Abstract

**Purpose:**

This study aimed to compare treatment outcomes for bloodstream infections (BSI) caused by a piperacillin/tazobactam (PIP/TAZ)-susceptible *E. coli* among three patient groups: BSI caused by ampicillin/sulbactam (AMP/SLB)-resistant isolates treated with PIP/TAZ, BSI caused by AMP/SLB-sensitive isolates treated with PIP/TAZ, and BSI caused by AMP/SLB-resistant isolates treated with another monotherapy.

**Methods:**

This retrospective study was conducted in two academic centres in Europe. Adult patients with *E. coli* BSI were screened from 2014 to 2020. Inclusion criteria were non-ESBL BSI and initial monotherapy for ≥ 72 h. To reduce the expected bias between the patient groups, propensity score matching was performed. The primary outcome was early treatment response after 72 h and required absence of SOFA score increase in ICU/IMC patients, as well as resolution of fever, leukocytosis, and bacteraemia.

**Results:**

Of the 1707 patients screened, 315 (18.5%) were included in the final analysis. Urinary tract infection was the most common source of BSI (54.9%). Monotherapies other than PIP/TAZ were cephalosporins (48.6%), carbapenems (34.3%), and quinolones (17.1%). Enhanced early treatment response rate was detected (*p* = 0.04) in patients with BSI caused by AMP/SLB-resistant isolates treated with another monotherapy (74.3%) compared to those treated with PIP/TAZ (57.1%), and was mainly driven by the use of cephalosporins and quinolones (*p* ≤ 0.03). Clinical success, 28-day mortality, and rate of relapsing BSI did not significantly differ between the groups.

**Conclusions:**

Our study suggests that initial use of PIP/TAZ may be associated with reduced early treatment response in *E. coli* BSI caused by AMP/SLB-resistant isolates compared to alternative monotherapies.

**Supplementary Information:**

The online version contains supplementary material available at 10.1007/s15010-023-02074-z.

## Introduction

*Escherichia coli (E. coli)* is the most common causative pathogen of gram-negative bloodstream infection (BSI) across Europe and other high-income countries [1–4]. The primary source of *E. coli* BSI are urogenital infections, followed by biliary and other intra-abdominal infections [1, 2, 5]. Less common sources include pneumonia, central-line-associated infections, postoperative wound infections, and infections with deep-seated focus [1, 2, 5, 6]. In recent years, rates of *E. coli* antibiotic resistance to aminopenicillins with beta-lactamase inhibitors are evidently increasing [2, 7]. Resistance to ampicillin/sulbactam (AMP/SLB) is associated with presence/hyperproduction of specific beta-lactamases, including plasmid-mediated TEM-1 beta-lactamase [8], inhibitor-resistant TEM (IRT) beta-lactamases [9, 10], other plasmid-mediated beta-lactamases (OXA-1, AmpC beta-lactamases and extended-spectrum beta-lactamases (ESBLs)) [11, 12] and chromosomal AmpC beta-lactamase [13–16].

Piperacillin/tazobactam (PIP/TAZ), composed of a ureidopenicillin with extended-spectrum activity and a beta-lactamase inhibitor, is a common first-line treatment option in severe infections, such as sepsis, pneumonia, intraabdominal infections, complicated urinary tract infections, gynaecological infections, and in patients with febrile neutropenia [17]. Despite its widespread use, PIP/TAZ resistance rates in invasive *E.* coli, including AMP/SLB-resistant isolates, remained below 10% in our two clinical centres in Austria and Germany. PIP/TAZ inhibits most class A beta-lactamases in the TEM, SHV and CTX-M series in vitro [18]. However, whether this in vitro susceptibility translates in comparable clinical outcomes when treating AMP/SLB-resistant *E. coli* strains with PIP/TAZ compared to AMP/SLB-sensitive strains has not been studied. This is particularly important, since antibiotic stewardship strategies usually foster a tailored treatment sparing carbapenems and 3rd generation cephalosporins. Therefore, this retrospective binational cohort study aimed to investigate the treatment responses to PIP/TAZ in patients with BSI caused by PIP/TAZ-susceptible, AMP/SLB-resistant *E. coli* (group *A*) compared to an alternative antibiotic monotherapy (group *C*). Additionally, we assessed treatment responses in patients with BSI caused by AMP/SLB-sensitive *E. coli* treated with PIP/TAZ (group *B*).

## Patients and methods

### Study population and study design

This retrospective cohort study was performed in two large academic centres in central Europe, the Jena University Hospital (JUH) in Germany and the Medical University of Vienna (MUW) in Austria. JUH is a 1400-bed academic hospital in the state Thuringia in Germany [19]. MUW is a 1700-bed academic hospital in the city and state Vienna in Austria.

After ethical approval, we included all adult patients with at least one blood culture (BC) positive for *E. coli* who received an antibiotic monotherapy for at least 72 h between January 2014 and June 2020. Exclusion criteria were polymicrobial bacteraemia, death within the first 72 h, treatment duration of initial antibiotic therapy < 72 h, ESBL-detection, AMP/SLB-sensitive *E. coli* not treated with PIP/TAZ, in vitro resistance to PIP/TAZ, no hospital admission, initial oral antibiotic treatment, or therapy without a curative goal.

### Microbiological BSI diagnostics

Two different BC systems were used in this study: the BD BACTEC^™^ FX system (BD Diagnostics, Heidelberg, Germany) at JUH and the BacT/Alert^®^ 3D system (bioMérieux, Marcy l’Etoile, France) at MUW. In general, one to three BC sets per blood draw were collected. The BC bottles were incubated for up to 5 days (JUH) or up to 7 days (MUW) in the respective BC system. Species identification was routinely performed the next day on culture-grown colonies by Vitek^®^ MS (MALDI-TOF (Matrix Assisted Laser Desorption IonizationTime-of-Flight) Mass Spectrometry, bioMérieux, Nürtingen, Germany) or MALDI Biotyper^®^ (MALDI-TOF MS, Bruker, Germany). Antimicrobial susceptibility testing was performed by Vitek-2 system (bioMérieux) and evaluated according to the EUCAST criteria. Only *E. coli* strains with an AMP/SLB or PIP/TAZ MIC of ≤ 8 mg/l were considered as susceptible to the respective substance. The ESBL status was primarily determined by Vitek-2 ESBL test at JUH and was evaluated by manual ESBL tests (ß LACTA^™^ test, BioRad; disc diffusion test with an ESBL inhibitor, Mast) at MUW.

### Data collection

The following data were collected from the patients’ medical records: demographic data, comorbidities, Pitt bacteraemia score, implanted devices, source of *E.coli* BSI (urinary, biliary and other intra-abdominal, vascular catheter-related, respiratory, skin/soft tissue, bone/joint), place of infection (hospital-acquired vs. other place), stay at an intensive care unit (ICU) or intermediate care (IMC) at the onset of BSI, fever > 38 °C, leukocytosis > 12 Gpt/l, Sequential Organ Failure Assessment (SOFA) score at baseline (day of sampling of the first positive BC), 72 h, and 14 days after sampling of the initial positive BC and start of active treatment, duration of BSI (if follow-up BCs were available), first- and second-line antibiotic therapy, duration of antibiotic treatment, source control, length of hospital stay and discharge mode (deceased, alive).

### Outcome analyses

The primary outcome of the study was early treatment response 72 h after the sampling of the initial positive BC and the start of active antimicrobial treatment. Early treatment response was defined as a composite measure and required all of the following parameters: absence of SOFA score increase in patients admitted to ICU or IMC, resolution of fever (temperature < 38 °C), leukocytosis (white blood cell count < 12 Gpt/l), and microbiologic resolution (no documented persistent bacteraemia ≥ 72 h) [20–22].

The secondary outcomes included the clinical success 14 days after sampling of the initial positive BC, the 28-day mortality rate, relapsing BSI within 60 days in patients with follow-up BCs and length of hospital stay.

### Sample size calculation

Sample size was based on a prior randomized controlled study in patients with BSI caused by ceftriaxone-resistant but PIP/TAZ-sensitive *E. coli* or *Klebsiella pneumoniae* [20]. In this study, clinical response was achieved at median day 3 in the PIP/TAZ group (IQR 1–5 days), indicating a proportion of 50% cases with a clinical response within 72 h. Considering these results, a sample size of 103 per group is needed to detect an absolute difference of 20% (early treatment response: 50% vs. 70%) on a significance level of 0.05 and 80% power with a two-sided Fisher’s exact test. Therefore, in this study the minimum total sample size is 309 (3 × 103) adult patients with *E. coli* BSI.

### Statistical analysis

To reduce the expected bias between the three patient groups, propensity score matching was performed. Propensity scores were calculated using a logistic regression model with patient group as the dependent variable, and variables potentially associated with the treatment decision and the clinical response (sex, age and centre) as the independent variables. In the first step, we calculated propensity scores for a subset containing cases from patient groups *A* and *C*, with group *A* considered the treatment group and group *C* as the control group. We matched group *C* to group *A* at a 1:1 ratio using the K-nearest-neighbors method without replacement. In the second step, group B was matched to the pre-matched group *A*, by repeating the matching procedure. Propensity score matching was performed using the R-package *MatchIt* (Ho, 2011) (supp. Fig. 1).

Baseline demographics were assessed using descriptive statistics. Continuous and discrete numerical variables were reported as median and interquartile range (IQR) or median and range. Categorical variables were reported as frequency and percentage. Fisher’s exact test was used to compare the primary and secondary endpoints between the three groups. *p* values were adjusted for multiple testing using the Holm–Bonferroni method. Univariate logistic regression was used to model the early clinical response based on the therapy group. A multiple logistic regression model was implemented to assess the influence of the antibiotic therapy on early clinical response in patients with BSI caused by AMP/SLB-resistant *E. coli.* This model included antibiotic therapy, ICU or IMC admission, centre, Pitt bacteraemia score, neutropenia, liver disease and BSI source. A two-sided significance level of 0.05 was applied in all models.

Statistical analysis was performed using R version 4.1.3 (R Core Team (2022). Vienna, Austria). The R packages ggplot2 (Wickham, 2016), ggpubr (Kassambara, 2020) and viridis (Garnier, 2021) were used for graphical representation of the data.

## Results

### Study cohort

**Fig. 1:**
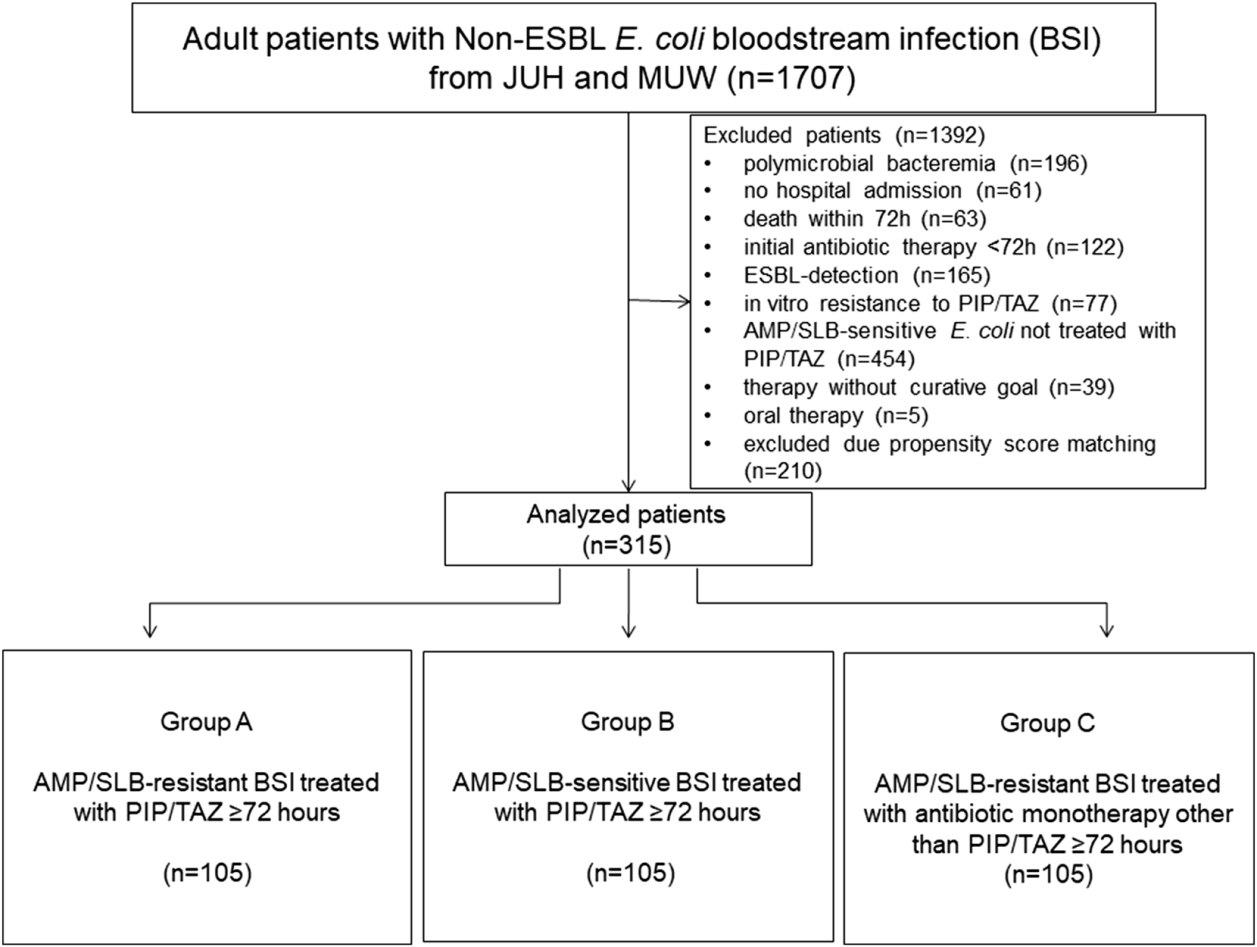
Flowchart diagram of the study population

A total of 1707 adult patients with *E. coli* BSI were screened (*n* = 867 from JUH and 840 from MUW), and 1392 patients (81.5%) were excluded. For detailed numbers please refer to Fig. 1. After propensity score matching, 315 patients were finally analysed (Jena *n* = 189, Vienna *n* = 126). Each group (group *A*, *B* and *C*) included 105 patients: 63 (60%) from Jena and 42 (40%) from Vienna. Detailed baseline characteristics of the patient cohort in Jena vs. Vienna are given in Supp. Table 1. In Jena, there were a higher rate of hospital-acquired infections (30.2% vs. 19.0%), a higher rate of multiple sources of BSI (17.5% vs. 0%) or pneumonia (11.1% vs. 1.6%), and a greater likelihood of implanted devices (54.5% vs. 26.2%) or being ICU or IMC patients (16.4% vs. 3.2%) at the onset of BSI. In addition, follow-up BCs (52.4% vs. 19.0%) were taken more frequently in Jena, but the duration of antibiotic therapy was shorter than in Vienna (median 9 vs. 14 days).

Table 1 includes detailed characteristics of propensity score matched groups. The median age was 65 years in group *A*, 69 years in group *B*, and 68 years in group *C*. At the onset of BSI, 280 patients (88.9%) were treated at a normal ward and 35 (11.1%) were admitted to an ICU or IMC, with the lowest rate in group *C* (8.6%). The predominant source of BSI was urinary tract infection (> 48% in all groups). Thirty-three patients (10.5%) had more than one source of infection, with the highest rate in group *C* (11.4%).

**Table 1:** Comparison of baseline characteristics and treatment parameters after propensity score matching between patients with bloodstream infection (BSI) caused by non-ESBL ampicillin/sulbactam (AMP/SLB)-resistant *E. coli* and initial treatment with piperacillin/tazobactam (PIP/TAZ) (group *A*), patients with BSI caused by AMP/SLB-sensitive *E. coli* and initial therapy with PIP/TAZ (group *B*), and patients with non-ESBL AMP/SLB-resistant *E. coli* and initial therapy with a monotherapy other than PIP/TAZ (group *C*)

	*A* (*n* = 105)	*B* (*n* = 105)	*C* (*n* = 105)
Centre, *n* (%)			
Jena	63 (60.0)	63 (60.0)	63 (60.0)
Vienna	42 (40.0)	42 (40.0)	42 (40.0)
Age in years, median (IQR)	65 (59–72)	69 (59–74)	68 (60–79)
Male sex, *n* (%)	55 (52.4)	55 (52.4)	55 (52.4)
BMI, median (IQR)	26.75 (23.52–30.9)	24.49 (22.3–28.64)	25.06 (23.15–27.78)
Normal ward, *n* (%)	90 (85.7)	94 (89.5)	96 (91.4)
Intensive care unit or intermediate care, *n* (%)	15 (14.3)	11 (10.5)	9 (8.6)
Charlson Comorbidity Index (CCI), median (IQR)	6 (4–8)	6 (3–8)	5 (3–8)
Pitt bacteraemia score, median (IQR)	1 (0–2)	0 (0–1)	1 (0–2)
Implanted devices^a^	41 (39)	44 (41.9)	51 (48.6)
Comorbidities, *n* (%)			
Haemato-oncological disease	40 (38.1)	22 (21)	27 (25.7)
Solid organ transplantation	13 (12.4)	18 (17.1)	15 (14.3)
Stem cell transplantation	6 (5.7)	3 (2.9)	6 (5.7)
Connective tissue disease	3 (2.9)	7 (6.7)	3 (2.9)
Chronic kidney disease	35 (33.3)	30 (28.6)	33 (31.4)
Diabetes mellitus	21 (20)	20 (19)	14 (13.3)
COPD	3 (2.9)	10 (9.5)	10 (9.5)
Vascular diseases	8 (7.6)	7 (6.7)	8 (7.6)
Chronic heart failure	31 (29.5)	20 (19)	31 (29.5)
Source of BSI, *n* (%)			
Primary bacteraemia	21 (20)	8 (7.6)	13 (12.4)
Vascular catheter-related BSI	3 (2.9)	6 (5.7)	4 (3.8)
Biliary/other intra-abdominal infection	21 (20)	19 (18.1)	16 (15.2)
Urinary tract infection	51 (48.6)	60 (57.1)	62 (59)
Pneumonia	9 (8.6)	8 (7.6)	6 (5.7)
Skin/soft tissue infection	0 (0)	0 (0)	2 (1.9)
Bone/joint infection	0 (0)	3 (2.9)	2 (1.9)
More than one source of BSI, *n* (%)	11 (10.5)	10 (9.5)	12 (11.4)
Nosocomial infection, *n* (%)	30 (28.6)	23 (21.9)	28 (26.7)
Surgical source control	9 (8.6)	10 (9.5)	10 (9.5)
Antibiotic dosages of initial therapy, *n* (%)			
Less than standard dose	0 (0)	1 (1)	15 (14.3)
Standard dose^b^	84 (80)	84 (80)	63 (60.6)
High dose^c^	21 (20)	20 (19)	27 (25.7)
Duration of initial therapy in days, median (IQR)	8 (5–10)	8 (6–11)	8 (5–11)
Number of patients with therapy change, *n*%	19 (18.1)	13 (12.4)	4 (3.8)
Duration of total therapy in days, median (IQR)	11 (8–16)	11 (8–14)	11 (8–15)

^a^Implanted devices include orthopedic devices, cardiac devices, and biliary/ureteral stents

^b^Standard daily doses were 13.5 g for piperacillin/tazobactam, 2 g for ceftriaxone, 3 g for cefotaxime or ceftazidime or aztreonam or meropenem, 1 g for ertapenem, 0.4 g for moxifloxacin, 0.5 g for levofloxacin, 0.8 g for ciprofloxacin

^c^High daily doses were 18 g for piperacillin/tazobactam, 4.5 g for cefuroxime, 4 g for ceftriaxone, 6 g for cefotaxime or ceftazidime or cefepime or meropenem, 1 g for levofloxacin, 1.2 g for ciprofloxacin

The median total antibiotic treatment duration was 11 days. Treatment change due to escalation from the initial antibiotic regimen to a carbapenem was highest with 16.1% in group *A*. Initial antibiotics used in group *C* were meropenem (*n* = 35), ceftriaxone (*n* = 24), cefotaxime (*n* = 12), ciprofloxacin (*n* = 14), cefuroxime (*n* = 6), ceftazidime (*n* = 6), cefepime (*n* = 3), and levofloxacin (*n* = 3). Ertapenem and moxifloxacin were used only once (*n* = 1). Overall, in Vienna, cephalosporins were utilized more frequently than in Jena, with a rate of 23.0% compared to 11.6%. Conversely, carbapenems were less commonly used in Vienna, with a rate of 4.8% compared to 15.9% in Jena. The rate of quinolones did not differ between the two centres, with a rate of 5.6% in Vienna and 5.8% in Jena.

### Primary and secondary outcomes

Detailed results of primary and secondary clinical endpoints are presented in Table 2. Overall, early treatment response was achieved in 207 of 315 patients (65.7%). No significant differences in early treatment responses were found between patients who received high antibiotic doses (39/68, 57.4%) and those with standard or low antibiotic doses (168/247, 68%, *p* = 0.113). The severity of disease had an impact on the antibiotic dosages and choice of initial treatment, as patients admitted to ICU or IMC had a higher proportion of high-dose treatment than patients on the normal ward (47.2% vs. 18.2%) and initial treatment of ICU or IMC patients was mainly PIP/TAZ (26/35, 74.2%), followed by carbapenems (6/35, 16.7%), cephalosporins (2/35, 5.7%), and quinolones (1/35, 2.9%).

**Table 2:** Comparison of primary and secondary outcomes between three different groups: patients with BSI caused by non-ESBL AMP/SLB-resistant *E. coli* and initial treatment with PIP/TAZ (group *A*), patients with BSI caused by AMP/SLB-sensitive *E. coli* and initial therapy with PIP/TAZ (group *B*), and patients with non-ESBL AMP/SLB-resistant *E. coli* BSI and initial therapy with an antibiotic monotherapy other than PIP/TAZ (group *C*)

	Descriptive statistics			Fisher’s exact test *p* value^a^		
	Group *A* (*n* = 105)	Group *B* (*n* = 105)	Group *C* (*n* = 105)	*A* vs. *B*	*A* vs. *C*	*B* vs. *C*
Early treatment response after 72 h, *n* (%)	60 (57.1)	69 (65.7)	78 (74.3)	0.456	0.04	0.456
Reason for treatment failure, *n* (%)						
Any increase in SOFA score	16 (15.2)	18 (17.1)	12 (11.4)			
Persistent fever	28 (26.7)	21 (20)	17 (16.2)			
Persistent leukocytosis	22 (21)	20 (19)	13 (12.4)			
Persistent bacteraemia ≥ 72 h	6 (5.7)	8 (7.6)	5 (4.8)			
Clinical response after 14 days^b^, *n* (%)	90 (87.4)	78 (83.9)	86 (84.3)	1	1	1
28-day mortality, *n* (%)	4 (3.8)	7 (6.7)	6 (5.7)	0.538	0.748	1
Relapsing BSI within 60 days in patients with follow-up blood cultures, *n*/*n* (%)	14/37 (37.8)	9/50 (18.0)	10/36 (27.8)	0.150	0.608	0.608
In vitro resistance to PIP/TAZ in relapsing BSI^c^, *n*/*n* (%)	10/14 (71.4)	4/9 (44.4)	3/3 (100)			
Length of hospital stay in days, median (range)	15 (9–29)	14 (10–23)	12 (8–23.5)	0.577	0.273	0.355

^a^Holm-adjusted

^b^Data are missing in two patients of Group *A*, in 12 patients of Group *B*, in 3 patients of Group *C*

^c^Resistance data are missing in 7 patients of Group *C*

The three patient groups were compared under statistical control of covariates. Centre and sex were matched exactly. The absolute standardized mean differences for age were 0.39 or lower. Detailed information on the propensity scores of the matched data is provided in Supp. Fig. 1. Using a two-sided Fisher’s exact test, we detected a significantly higher early treatment response rate of 74.3% in group *C*, compared to group *A*’s response rate of 57.1% (*p* = 0.04). No significant difference in response rate was found between group *C* and group *B* (65.7%, *p* = 0.456), or between group *A* and *B* (*p* = 0.456).

Univariate logistic regression revealed a significantly higher odds ratio of an early treatment response in group *C* (OR = 2.17, *p* = 0.009) compared to group *A*, corresponding to an average marginal effect (AME) of 17.14% (CI = 4.51–29.77%).

**Fig. 2:**
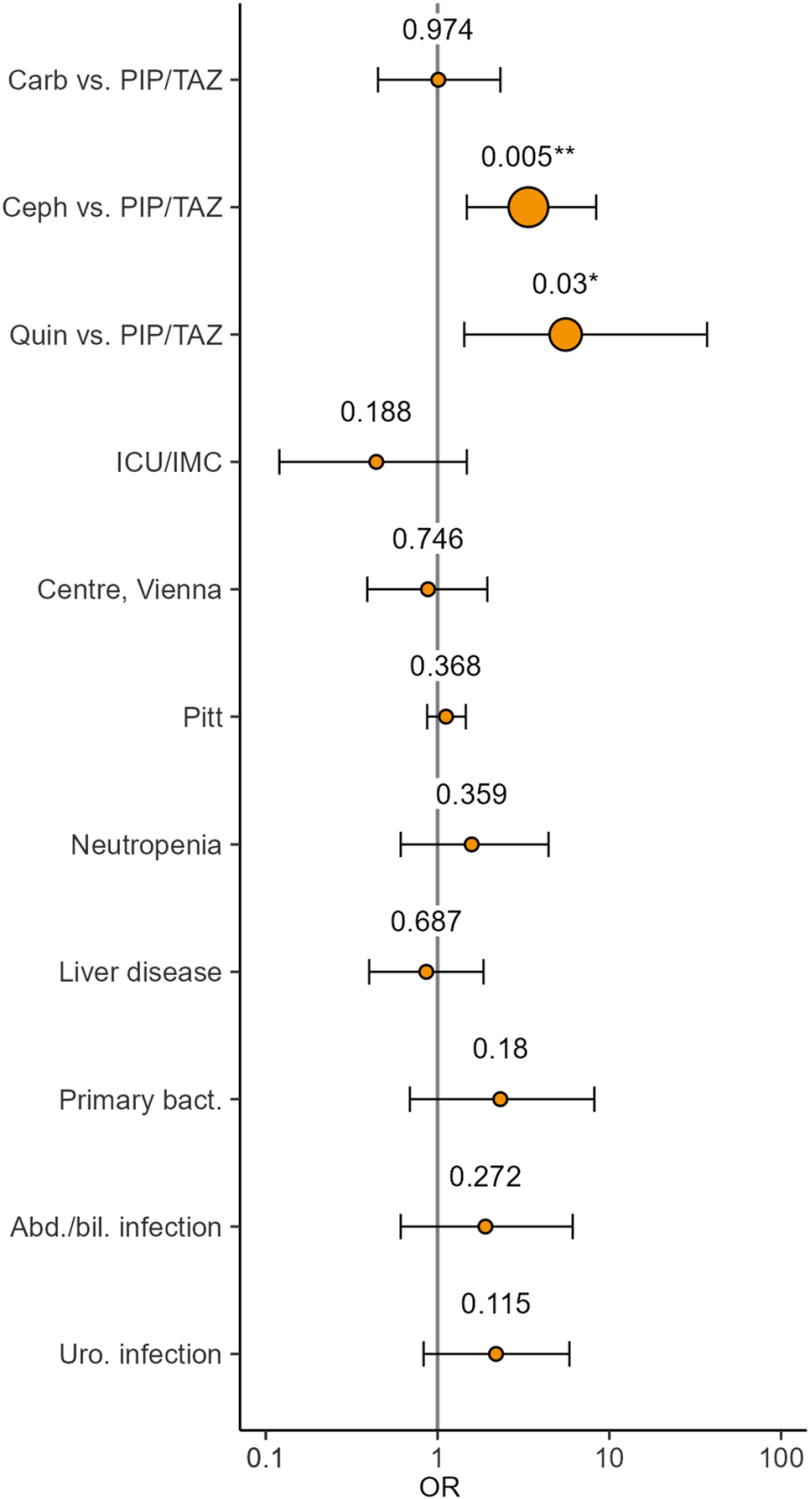
Forestplot for multivariable logistic regression for early treatment response after 72 h on patients with BSI caused by non-ESBL AMP/SLB-resistant *E. coli.* The model includes antibiotic therapy, ICU/IMC, centre, Pitt bacteraemia score, neutropenia, liver disease and source of BSI (primary bacteraemia, abdominal/biliary infection, and urogenital infection compared to other sources)

In a multivariable logistic regression model including antibiotic therapy, ICU or IMC admission, centre, Pitt bacteraemia score, neutropenia, liver disease and source of bacteraemia as covariates, cephalosporins (OR = 3.38, CI = 1.48–8.39, *p* = 0.005) and quinolones (OR = 5.57, CI = 1.43–37.08, *p* = 0.03) were identified as positive predictors of early treatment response in patients with BSI caused by AMP/SLB-resistant *E. coli*, corresponding to average marginal effects of 24% (CI 9.24–38.76%) and 30.27% (CI 11.73–48.81), respectively (Fig. 2, Suppl. Table 2).

Early treatment failure was mainly attributed to persistent fever, followed by persistent leukocytosis, and any increase in SOFA score. Notably, the rate of persistent bacteraemia ≥ 72 h ranged between 4.8 and 7.6%.

**Fig. 3:**
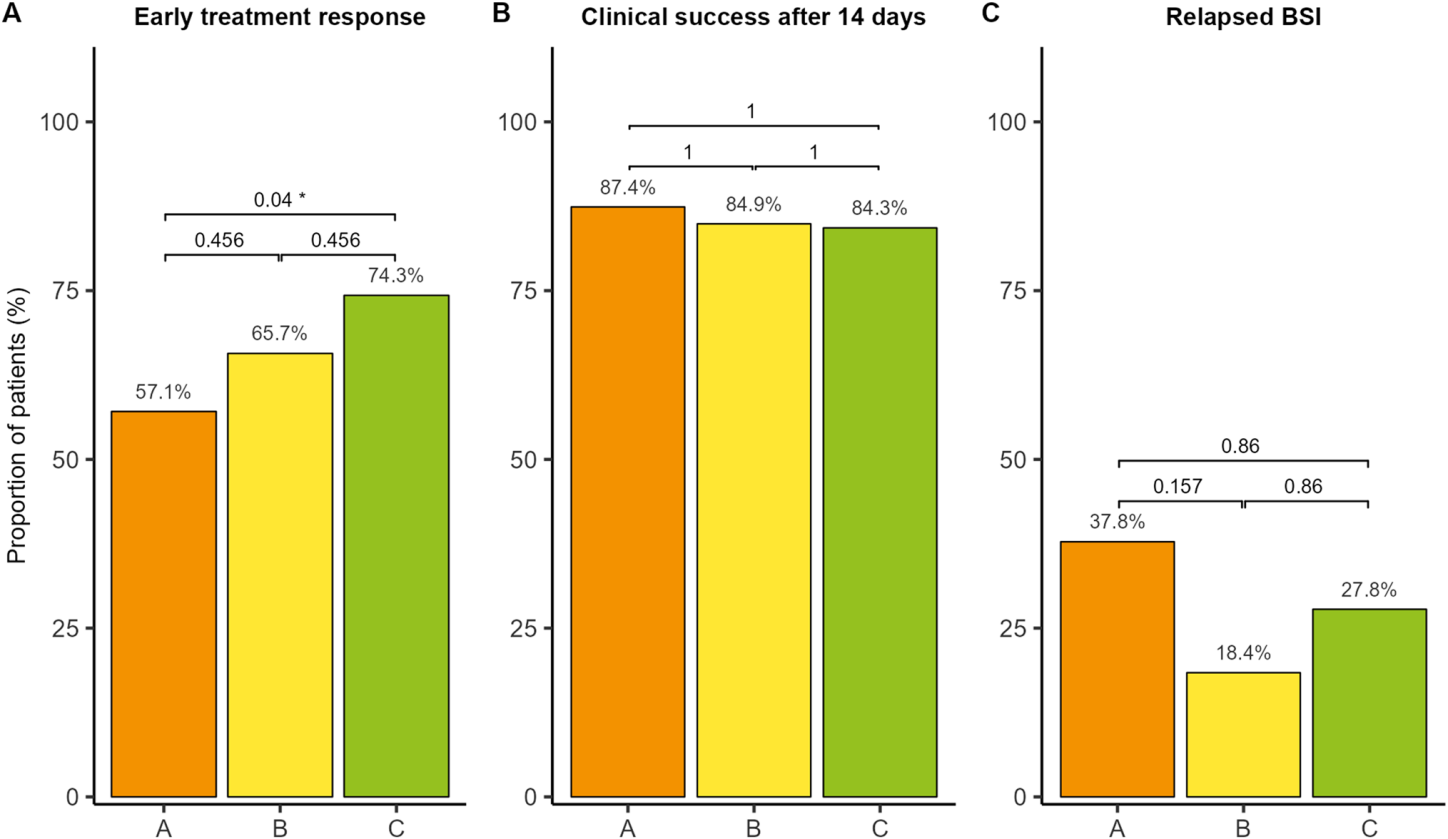
Comparison of patients with bloodstream infections (BSI) caused by non-ESBL AMP/SLB-resistant *E. coli* and initial treatment with PIP/TAZ (group *A*) vs. patients with BSI caused by AMP/SLB-sensitive *E. coli* and initial therapy with PIP/TAZ (group *B*) vs. patients with BSI caused by non-ESBL AMP/SLB-resistant *E. coli* and initial antibiotic monotherapy other than PIP/TAZ (group *C*) regarding **A** early treatment response after 72 h, **B** clinical success after 14 days and **C** relapsing BSI in patients with follow-up blood cultures within 60 days (group *A* *n* = 37, group *B* *n* = 50, group *C* *n* = 36). Brackets indicate the adjusted *p* values (Holm-correction) of a two-sided Fisher’s exact test

As shown in Fig. 3, there was no significant difference in clinical success between all groups after 14 days (*p* = 1.0). Overall, patients had a low 28-day mortality rate: 3.8% in group *A*, 6.7% in group *B*, and 5.7% in group *C* (*p* ≥ 0.538). The median length of hospital stay was 12–15 days.

Out of 123 patients with follow-up BCs, 26.8% experienced relapsing BSI. The rate of relapsing BSI was numerically higher in patients with initial AMP/SLB-resistant BSI (group *A* 14/37 (37.8%), group *C* 10/36 (27.8%)) compared to those with initial AMP/SLB-sensitive BSI (group *B*: 9/50 (18%)). However, the differences between the groups were not statistically significant (*p* ≥ 0.15). In vitro resistance to PIP/TAZ was detected in 17/26 patients with relapsing BSI. In vitro resistance to PIP/TAZ (MIC > 16 mg/l) was high in patients with relapsing BSI, with 10/14 in group *A*, 3/3 in group *C* (missing data on PIP/TAZ in vitro resistance in 7 patients in group *C*), and 4/9 in group *B*.

## Discussion

To our knowledge, this bicentre cohort study is the first to compare the early treatment response to PIP/TAZ in non-ESBL *E. coli* BSI caused by AMP/SLB-resistant and sensitive isolates with in vitro susceptibility to PIP/TAZ. The overall treatment response within 72 h of positive BC and start of active antibiotic treatment was achieved in approximately two-thirds of all patients, with nearly 90% of patients treated in the normal ward at the time of BSI. The 28-day mortality rate in this study was lower (< 7%) than in a systematic review of literature by Bonten et al*.* that included 210 studies on reported *E. coli* BSI in adults of high-income countries. There, the pooled estimated case fatality rate was 12.4% [1]. The lower mortality rate in present study was also due to the fact that we excluded patients who died within the first 72 h. The majority of those patients had an advanced underlying malignant or severely decompensated cardiac disease and refused intensive life-saving measures.

Interestingly, our study did not reveal any significant differences in early treatment response, clinical success, and 28-day mortality rate between patients with BSI caused by AMP/SLB-sensitive and AMP/SLB-resistant *E. coli* who were treated with PIP/TAZ. However, we did observe a higher early treatment response in patients with BSI caused by AMP/SLB-resistant *E. coli* who were treated with another monotherapy (48.6% cephalosporins, 34.3% carbapenems, 17.1% quinolones) than those treated with PIP/TAZ (74.3% vs. 57.1%). As only patients with non-ESBL *E. coli* isolates tested susceptible to PIP/TAZ (MIC ≤ 8 mg/l) in vitro were included in the study, this monotherapy should also be an adequate treatment option. However, in patients with BSI due to an AMP/SLB-resistant *E. coli,* treatment escalation from PIP/TAZ to a carbapenem was performed more frequently than in patients with initial cephalosporin or quinolone therapy (16.1% vs. 4.3%). Interestingly, not an initial therapy with carbapenems but use of cephalosporins and quinolones were both significantly associated with an enhanced early treatment response. These antibiotics were mainly but not exclusively used in the normal ward and admission to ICU or IMC was not identified as an independent negative predictor of early treatment response in the multivariable logistic regression model.

The current study found that the rate of relapse for BSI in patients with follow-up BCs was 10–20% higher in those with BSI due to an initial AMP/SLB-resistant *E. coli* (Group *A*: 37.8%; Group *C*: 27.8%) compared to those with initial AMP/SLB-sensitive isolates (Group *B*: 18%). This indicates that patients with an initial AMP/SLB-resistant *E. coli* are more likely to experience a relapse than those with initial AMP/SLB-sensitive isolates. In vitro susceptibility to PIP/TAZ may not always correlate with clinical efficacy, as antibiotic heteroresistance or selection of IRT TEM beta-lactamases by the administration of betalactam/beta-lactamase inhibitor combinations [23] have been linked to treatment failures in previous studies [24, 25]. In addition, altered pharmacokinetics due to the pathophysiologic changes associated with critical illness and decreased antibiotic sensitivity to the pathogen [26] may also contribute to inadequate antibiotic efficacy. In the past, some clinical studies had suggested that there may be issues with the in vitro susceptibility testing of PIP/TAZ using Vitek 2. In a retesting of the clinical BSI isolates of the Merino trial by microdilution testing, the authors Henderson et al*.* found that 6% of isolates were resistant to PIP/TAZ [27]. Excluding those isolates with PIP/TAZ MIC > 16 mg/l, the absolute risk difference in 30-day mortality between PIP/TAZ and meropenem in ceftriaxone-resistant *E. coli* and *Klebsiella pneumoniae* BSI was reduced from 9 to 5%. Furthermore, it is worth noting that until 2020, the last year of our recruiting period, EUCAST and the national antibiotic sensitivity committee in Germany recommended piperacillin/tazobactam 4.5 g given three times daily as the standard dose. However, EUCAST now recommends a higher dose of piperacillin/tazobactam (4.5 g given four times daily by 30-min infusion) or prolonged infusion (4.5 g given three time daily by 4 h extended infusion) for bloodstream infections. As a result, our data indicates that most patients at the normal ward received the lower standard dose which may have impacted the outcomes of our study. It is important to consider these factors when interpreting our findings.

Our study has several limitations. First, the study was retrospective, and there were a number of differences in the microbiological testing workflow and evaluation of the ESBL status, but non-ESBL beta-lactamases including presence of AmpC beta-lactamases were not reported to the clinicians in both centres. Therefore, the mechanism of AMP/SLB resistance is unknown. Second, therapeutic drug monitoring (TDM) was not routinely performed, even in ICU patients receiving prolonged or continuous infusion. However, most patients were non-ICU patients and even in a recent randomized multicenter study with 254 patients, the authors Hagel et al*.* did not identify a significant clinical and microbiological benefit for TDM-guided therapy in patients with sepsis and continuous infusion of PIP/TAZ [28]. Third, we cannot exclude that repeated BSI episodes after end of treatment were true relapse events vs. new infections with mainly resistant strains, as isolates were not available for molecular typing.

In conclusion, our study suggests that initial use of PIP/TAZ in adult patients with non-ESBL *E. coli* BSI caused by an AMP/SLB-resistant isolate may be associated with a lower early treatment response rate compared to other monotherapies, particularly cephalosporins and quinolones. However, the results of this study are not sufficient yet to give a strong recommendation to avoid PIP/TAZ in patients with *E. coli* BSI caused by AMP/SLB-resistant isolates as clinical success after 14 days, 28-day mortality rate, and rate of relapsing BSI did not significantly differ between the treatment groups.

### Supplementary Information

Below is the link to the electronic supplementary material.

Supplementary file1 (DOCX 300 KB)

## Data Availability

The data sets used and/or analyzed during the current study are available upon reasonable request from the corresponding author.

## References

[1] Bonten M, Johnson JR, van den Biggelaar AHJ, Georgalis L, Geurtsen J, de Palacios PI (2021). Epidemiology of *Escherichia coli* bacteremia: a systematic literature review. Clin Infect Dis.

[2] Kern WV, Rieg S (2020). Burden of bacterial bloodstream infection-a brief update on epidemiology and significance of multidrug-resistant pathogens. Clin Microbiol Infect.

[3] Schöneweck F, Schmitz RPH, Rißner F, Scherag A, Löffler B, Pletz MW (2021). The epidemiology of bloodstream infections and antimicrobial susceptibility patterns in Thuringia, Germany: a five-year prospective, state-wide surveillance study (AlertsNet). Antimicrob Resist Infect Control.

[4] Verway M, Brown KA, Marchand-Austin A, Diong C, Lee S, Langford B (2022). Prevalence and mortality associated with bloodstream organisms: a population-wide retrospective cohort study. J Clin Microbiol.

[5] de Lastours V, Laouénan C, Royer G, Carbonnelle E, Lepeule R, Esposito-Farèse M (2020). Mortality in *Escherichia coli* bloodstream infections: antibiotic resistance still does not make it. J Antimicrob Chemother.

[6] Forstner C, Patchev V, Rohde G, Rupp J, Witzenrath M, Welte T (2020). Rate and predictors of bacteremia in afebrile community-acquired pneumonia. Chest.

[7] Vihta KD, Stoesser N, Llewelyn MJ, Quan TP, Davies T, Fawcett NJ (2018). Trends over time in *Escherichia coli* bloodstream infections, urinary tract infections, and antibiotic susceptibilities in Oxfordshire, UK, 1998–2016: a study of electronic health records. Lancet Infect Dis.

[8] Wu PJ, Shannon K, Phillips I (1994). Effect of hyperproduction of TEM-1 beta-lactamase on in vitro susceptibility of *Escherichia coli* to beta-lactam antibiotics. Antimicrob Agents Chemother.

[9] Sirot D, Chanal C, Henquell C, Labia R, Sirot J, Cluzel RA (1994). Clinical isolates of *Escherichia coli* producing multiple TEM mutants resistant to beta-lactamase inhibitors. J Antimicrob Chemother.

[10] Martín O, Valverde A, Morosini MI, Rodríguez-Domínguez M, Rodríguez-Baños M, Coque TM (2010). Population analysis and epidemiological features of inhibitor-resistant-TEM-beta-lactamase-producing *Escherichia coli* isolates from both community and hospital settings in Madrid, Spain. J Clin Microbiol.

[11] Zhou XY, Bordon F, Sirot D, Kitzis MD, Gutmann L (1994). Emergence of clinical isolates of *Escherichia coli* producing TEM-1 derivatives or an OXA-1 beta-lactamase conferring resistance to beta-lactamase inhibitors. Antimicrob Agents Chemother.

[12] Kaye KS, Gold HS, Schwaber MJ, Venkataraman L, Qi Y, De Girolami PC (2004). Variety of beta-lactamases produced by amoxicillin-clavulanate-resistant *Escherichia coli* isolated in the northeastern United States. Antimicrob Agents Chemother.

[13] Waltner-Toews RI, Paterson DL, Qureshi ZA, Sidjabat HE, Adams-Haduch JM, Shutt KA (2011). Clinical characteristics of bloodstream infections due to ampicillin-sulbactam-resistant, non-extended- spectrum-beta-lactamase-producing *Escherichia coli* and the role of TEM-1 hyperproduction. Antimicrob Agents Chemother.

[14] Rodríguez-Baño J, Oteo J, Ortega A, Villar M, Conejo MC, Bou G (2013). Epidemiological and clinical complexity of amoxicillin-clavulanate-resistant *Escherichia coli*. J Clin Microbiol.

[15] Alvarez M, Tran JH, Chow N, Jacoby GA (2004). Epidemiology of conjugative plasmid-mediated AmpC beta-lactamases in the United States. Antimicrob Agents Chemother.

[16] Stapleton P, Wu PJ, King A, Shannon K, French G, Phillips I (1995). Incidence and mechanisms of resistance to the combination of amoxicillin and clavulanic acid in *Escherichia coli*. Antimicrob Agents Chemother.

[17] Perry CM, Markham A (1999). Piperacillin/tazobactam: an updated review of its use in the treatment of bacterial infections. Drugs.

[18] Tooke CL, Hinchliffe P, Bragginton EC, Colenso CK, Hirvonen VHA, Takebayashi Y (2019). β-Lactamases and β-Lactamase inhibitors in the 21st century. J Mol Biol.

[19] Bahrs C, Kimmig A, Weis S, Ankert J, Hagel S, Maschmann J (2022). Prospective surveillance study in a 1400-bed university hospital: COVID-19 exposure at home was the main risk factor for SARS-CoV-2 point seroprevalence among hospital staff. Transbound Emerg Dis.

[20] Harris PNA, Tambyah PA, Lye DC, Mo Y, Lee TH, Yilmaz M (2018). Effect of piperacillin-tazobactam vs meropenem on 30-day mortality for patients with *E. coli* or Klebsiella pneumoniae bloodstream infection and ceftriaxone resistance: a randomized clinical trial. JAMA.

[21] Paul M, Daikos GL, Durante-Mangoni E, Yahav D, Carmeli Y, Benattar YD (2018). Colistin alone versus colistin plus meropenem for treatment of severe infections caused by carbapenem-resistant Gram-negative bacteria: an open-label, randomised controlled trial. Lancet Infect Dis.

[22] Herrmann L, Kimmig A, Rödel J, Hagel S, Rose N, Pletz MW (2021). Early treatment outcomes for bloodstream infections caused by potential AmpC beta-lactamase-producing enterobacterales with focus on piperacillin/tazobactam: a retrospective cohort study. Antibiotics (Basel).

[23] Nicoloff H, Hjort K, Levin BR, Andersson DI (2019). The high prevalence of antibiotic heteroresistance in pathogenic bacteria is mainly caused by gene amplification. Nat Microbiol.

[24] Blazquez J, Baquero MR, Canton R, Alos I, Baquero F (1993). Characterization of a new TEM-type beta-lactamase resistant to clavulanate, sulbactam, and tazobactam in a clinical isolate of *Escherichia coli*. Antimicrob Agents Chemother.

[25] Henquell C, Sirot D, Chanal C, De Champs C, Chatron P, Lafeuille B (1994). Frequency of inhibitor-resistant TEM beta-lactamases in *Escherichia coli* isolates from urinary tract infections in France. J Antimicrob Chemother.

[26] Huttner A, Harbarth S, Hope WW, Lipman J, Roberts JA (2015). Therapeutic drug monitoring of the β-lactam antibiotics: what is the evidence and which patients should we be using it for?. J Antimicrob Chemother.

[27] Henderson A, Paterson DL, Chatfield MD, Tambyah PA, Lye DC, De PP (2021). Association between minimum inhibitory concentration, beta-lactamase genes and mortality for patients treated with piperacillin/tazobactam or meropenem from the MERINO study. Clin Infect Dis.

[28] Hagel S, Bach F, Brenner T, Bracht H, Brinkmann A, Annecke T (2022). Effect of therapeutic drug monitoring-based dose optimization of piperacillin/tazobactam on sepsis-related organ dysfunction in patients with sepsis: a randomized controlled trial. Intensive Care Med.

