# Supplementary material for: Early treatment response to piperacillin/tazobactam in patients with bloodstream infections caused by non-ESBL ampicillin/sulbactam-resistant Escherichia coli: a binational cohort study
Source: Infection. 2023 Jul 18;51(6):1749–58. doi: 10.1007/s15010-023-02074-z (PMC10665230; doi:10.1007/s15010-023-02074-z)
Supplement: Supplementary file 1 — Supplementary file1 (DOCX 300 KB) [file 15010_2023_2074_MOESM1_ESM.docx]

**Supp. Table 1. Baseline** characteristics of the study cohort stratified for the participating centre (Jena versus Vienna)

|  | **Jena (n = 189)** | **Vienna (n = 126)** |
| --- | --- | --- |
| *Initial antibiotic therapy, n (%)* |  |  |
| Piperacillin/tazobactam | 126 (66.7) | 84 (66.7) |
| Cephalosporins | 22 (11.6) | 29 (23.0) |
| Carbapenems | 30 (15.9) | 6 (4.8) |
| Quinolones | 11 (5.8) | 7 (5.6) |
| Age in years, median (IQR) | 66 (56 - 74) | 69 (61 - 75.8) |
| Male gender, n (%) | 90 (47.6) | 75 (59.5) |
| BMI, median (IQR) | 26.5 (23.3 - 30.6) | 24.6 (22.5 - 27.0) |
| Normal ward, n (%) | 158 (83.6) | 122 (96.8) |
| Intensive care unit or intermediate care, n (%) | 31 (16.4) | 4 (3.2) |
| Charlson Comorbidity Index (CCI), median (IQR) | 6 (3 - 8) | 6 (4 - 8) |
| Pitt bacteraemia score, median (IQR) | 1 (0 - 1) | 2 (0 - 2) |
| Implanted devices^1^ | 103 (54.5) | 33 (26.2) |
| *Comorbidities, n (%)* |  |  |
| Haemato-oncological disease | 45 (23.8) | 44 (34.9) |
| Solid organ transplantation | 24 (12.7) | 22 (17.5) |
| Stem cell transplantation | 5 (2.6) | 10 (7.9) |
| Connective tissue disease | 6 (3.2) | 7 (5.6) |
| Chronic kidney disease | 50 (26.5) | 48 (38.1) |
| Diabetes mellitus | 39 (20.6) | 16 (12.7) |
| COPD | 19 (10.1) | 4 (3.2) |
| Vascular diseases | 8 (4.2) | 15 (11.9) |
| Chronic heart failure | 46 (24.3) | 36 (28.6) |
| *Source of BSI, n (%)* |  |  |
| Primary bacteraemia | 14 (7.4) | 28 (22.2) |
| Vascular catheter-related BSI | 12 (6.3) | 1 (0.8) |
| Biliary/intra-abdominal infection | 33 (17.5) | 23 (18.3) |
| Urinary tract infection | 104 (55) | 69 (54.8) |
| Pneumonia | 21 (11.1) | 2 (1.6) |
| Skin/soft tissue infection | 1 (0.5) | 1 (0.8) |
| Bone or joint infection | 4 (2.1) | 1 (0.8) |
| More than one source of BSI, n (%) | 33 (17.5) | 0 (0) |
| Nosocomial infection, n (%) | 57 (30.2) | 24 (19) |
| Follow-up blood cultures, n (%) | 99 (52.4) | 24 (19.0) |
| Surgical source control | 13 (6.9) | 16 (12.7) |
| *Antibiotic dosages of initial therapy, n (%)* |  |  |
| Less than standard dose | 10 (5.3) | 6 (4.8) |
| Standard dose^2^ | 143 (75.7) | 88 (70.4) |
| High dose^3^ | 36 (19) | 32 (25.4) |
| Duration of initial therapy in days, median (IQR) | 7 (5 - 9) | 11 (7 - 15) |
| Number of patients with therapy change, n% | 25 (13.2) | 11 (8.7) |
| Duration of total therapy in days, median (IQR) | 9 (8 - 12) | 14 (10 - 17.8) |

BSI = bloodstream infection

^1^ Implanted devices include orthopedic devices, cardiac devices, and biliary/ureteral stents.

^2^ Standard daily doses were 13.5g for piperacillin/tazobactam, 2g for ceftriaxone, 3g for cefotaxime or ceftazidime or aztreonam or meropenem, 1g for ertapenem, 0.4g for moxifloxacin, 0.5g for levofloxacin, 0.8g for ciprofloxacin.

^3^ High daily doses were 18g for piperacillin/tazobactam, 4.5g for cefuroxime, 4g for ceftriaxone, 6g for cefotaxime or ceftazidime or cefepime or meropenem, 1g for levofloxacin, 1.2 g for ciprofloxacin.

**Supp. Figure 1.** Distance (A) and absolute standardized mean difference (B) after propensity score matching by centre, sex and age.


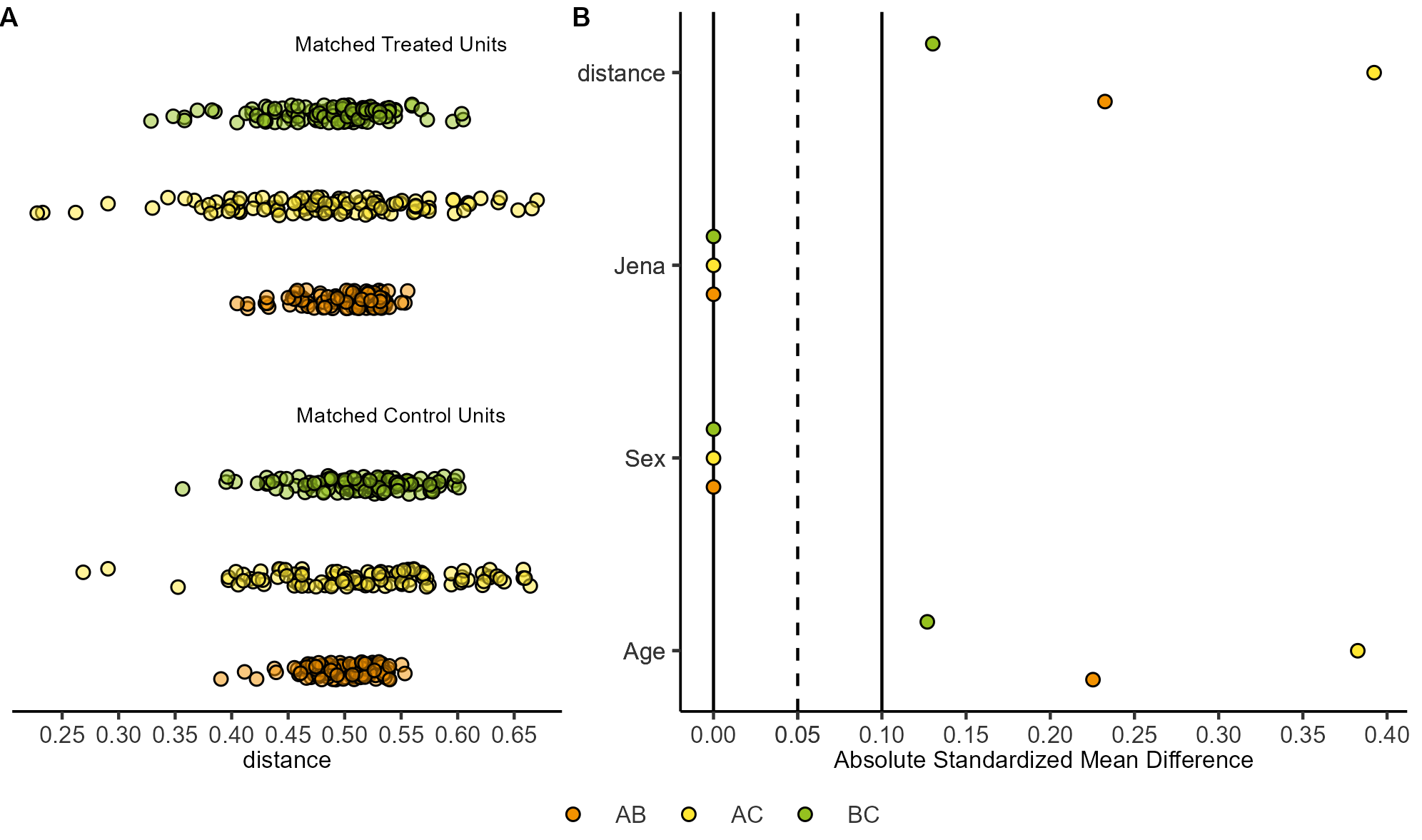


**Suppl. Table 2.** – Multivariable logistic regression model for early treatment response after 72 hours on patients with BSI caused by non-ESBL AMP/SLB-resistant *E. coli*

|  | **OR** | **CI 2.5** | **CI 97.5** | **p-value** | **AME (%)** | **CI 2.5 (%)** | **CI 97.5 (%)** |
| --- | --- | --- | --- | --- | --- | --- | --- |
| Carbapenems vs. PIP/TAZ | 1.01 | 0.45 | 2.32 | 0.974 | 0.32 | -18.93 | 19.58 |
| Cephalosporins vs. PIP/TAZ | 3.38 | 1.48 | 8.39 | 0.005 | 24 | 9.24 | 38.76 |
| Quinolones vs. PIP/TAZ | 5.57 | 1.43 | 37.08 | 0.03 | 30.27 | 11.73 | 48.81 |
| ICU/IMC | 0.44 | 0.12 | 1.48 | 0.188 | -16.51 | -40.69 | 7.67 |
| Centre, Vienna | 0.88 | 0.39 | 1.95 | 0.746 | -2.64 | -18.56 | 13.28 |
| Pitt bacteraemia score | 1.12 | 0.87 | 1.46 | 0.368 | 2.31 | -2.68 | 7.3 |
| Neutropenia | 1.58 | 0.61 | 4.43 | 0.359 | 9.25 | -10.35 | 28.85 |
| Liver disease^1^ | 0.86 | 0.4 | 1.85 | 0.687 | -3.13 | -18.36 | 12.09 |
| Primary bacteraemia^2^ | 2.32 | 0.69 | 8.18 | 0.18 | 15.51 | -4.44 | 35.46 |
| Abdominal/biliary infection^2^ | 1.9 | 0.61 | 6.12 | 0.272 | 11.96 | -7.35 | 31.27 |
| Urogenital infection^2^ | 2.19 | 0.83 | 5.86 | 0.115 | 15.81 | -3.31 | 34.93 |

^1^ Compared to patients without liver disease

^2^ Compared to patients with other BSI sources
